# Supplementary material for: Changes in dynamic transitions between integrated and segregated states underlie visual hallucinations in Parkinson’s disease
Source: Commun Biol. 2022 Sep 8;5:928. doi: 10.1038/s42003-022-03903-x (PMC9458713; doi:10.1038/s42003-022-03903-x)
Supplement: Supplementary file 2 — Supplementary Information [file 42003_2022_3903_MOESM2_ESM.pdf]

Supplementary Material: Changes in dynamic transition between integrated and segregated states underlie visual hallucinations in Parkinson's disease

Angeliki Zarkali, Andrea Luppi, Emanuel Stamatakis, Susanne Reeves, Peter McColgan, Louise-Ann Leyland, Andrew J. Lees, Rimona S. Weil

Table of Contents

Supplementary Table 1. Image quality metrics 2

Supplementary Table 2. List of selected genes encoding neurotransmitter receptors 3

Supplementary Figure 1. Optimal number of clusters 4

Supplementary Figure 2. Differences between Integrated and Segregated functional connectivity states 5

Supplementary Figure 3. Mean minimal control energy to maintain and transition between sub-states 6

Supplementary Figure 4. Altered temporal properties of dynamic functional connectivity in patients with Parkinson's and visual hallucinations:Replication with Schaeffer Parcellation 454 8

URLs 9

Supplementary Table 1. Image quality metrics

Metric	Controls n=32	PD non VH n=75	PD-VH n=16	Statistic
**Mean framewise displacement**	0.17 (0.09)	0.18 (0.12)	0.23 (0.15)	r^2=0.013 p=0.177
**Coefficient of joint variation**	0.69 (0.26)	0.67 (0.20)	0.68 (0.26)	r^2=-0.017 p=0.921
**Entropy focus criterion**	0.59 (0.02)	0.60 (0.02)	0.59 (0.02)	r^2=0.013 p=0.045*
**Total signal to noise ratio**	1.89 (0.17)	1.85 (0.18)	1.82 (0.11)	r^2=0.002 p=0.338

Results presented are mean (sd).
** No statistically significant difference found between any comparisons in post-hoc testing (Tukey).*

Supplementary Table 2. List of selected genes encoding neurotransmitter receptors

Gene symbol	Receptor (subunit) name
**Norepinephrine**	
ADRA1A	Alpha-1A adrenergic receptor
ADRA1B	Alpha-1B adrenergic receptor
ADRA1D	Alpha-1D adrenergic receptor
ADRA2A	Alpha-2A adrenergic receptor
ADRA2C	Alpha-2C adrenergic receptor
**Acetylcholine**	
CHRM1	Muscarinic Acetylcholine Receptor M1
CHRM2	Muscarinic Acetylcholine Receptor M2
CHRM3	Muscarinic Acetylcholine Receptor M3
CHRM4	Muscarinic Acetylcholine Receptor M4
CHRM5	Muscarinic Acetylcholine Receptor M5
CHRNA2	Nicotinic Cholinergic Receptor (Alpha 2)
CHRNA3	Nicotinic Cholinergic Receptor (Alpha 3)
CHRNA4	Nicotinic Cholinergic Receptor (Alpha 4)
CHRNA6	Nicotinic Cholinergic Receptor (Alpha 6)
CHRNA7	Nicotinic Cholinergic Receptor (Alpha 7)
CHRNA10	Nicotinic Cholinergic Receptor (Alpha 10)
CHRNB1	Nicotinic Cholinergic Receptor (Beta 1)
CHRNB2	Nicotinic Cholinergic Receptor (Beta 2)
**Dopamine**	
DRD1	Dopamine Receptor D1
DRD2	Dopamine Receptor D2
DRD4	Dopamine Receptor D4
**Serotonin**	
HTR1A	5-Hydroxytryptamine Receptor 1A, G protein-coupled
HTR1E	5-Hydroxytryptamine Receptor 1E, G protein-coupled
HTR1F	5-Hydroxytryptamine Receptor 1F, G protein-coupled
HTR2A	5-Hydroxytryptamine Receptor 2A, G protein-coupled
HTR2C	5-Hydroxytryptamine Receptor 2C, G protein-coupled
HTR3B	5-Hydroxytryptamine Receptor 3B, ionotropic
HTR3C	5-Hydroxytryptamine Receptor 3C, ionotropic
HTR4	5-Hydroxytryptamine Receptor 4, G protein-coupled
HTR5A	5-Hydroxytryptamine Receptor 5A, G protein-coupled
HTR7	5-Hydroxytryptamine Receptor 7, G protein-coupled

ADRA2B, CHRNA1, CHRNA5, CHRNA9, CHRNB3, CHRND, CHRNE, DRD3, DRD5, HTR1B, HTR1D, HTR3D, HTR3E, HTR5BP, HTR6 were not included in the analysis as they failed preprocessing steps.

Supplementary Figure 1. Optimal number of clusters

For all participants the Calinski-Harabasz score was calculated for 2-7 clusters. Cluster scores were ranked with best score (highest) ranked 1 and worse (lowest) score ranked 6 per participant. Overall across our cohort, N=2 clusters was best performing. Line: mean, Shaded area: 95% confidence intervals)

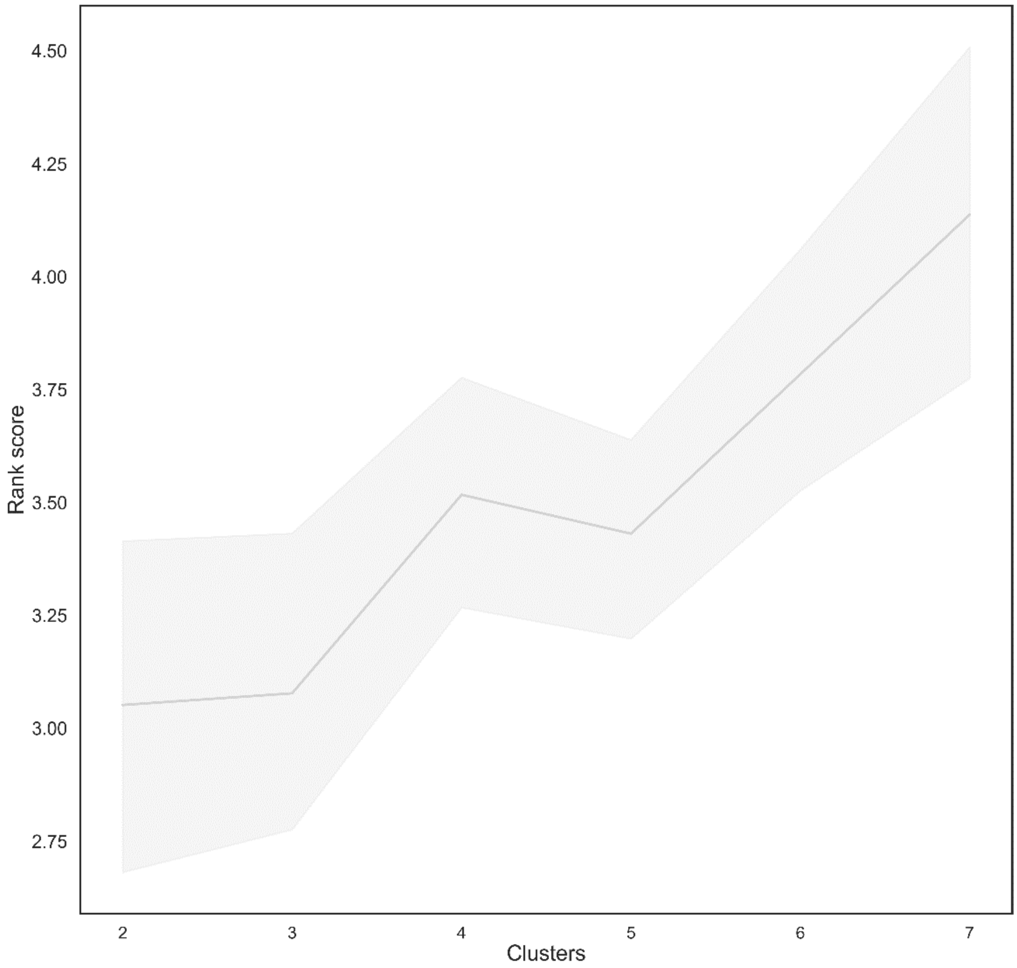

Supplementary Figure 2. Differences between Integrated and Segregated functional connectivity states

A. Difference between Integrated and Segregated states. Connectivity matrix of the difference between Integrated and Segregated States. Network based statistics was performed across all participants, with Integrated vs Segregated state as contrast of interest. The visualised connectivity matrix shows all connections that are statistically different (FWE-corrected) between the two states; all connections showed reduced connectivity strength in the Segregated compared to the Integrated state. No statistically significant results were seen in the opposite direction.

B. Regional node distribution in the differences between Integrated and Segregated state. Nodes that show higher number of connections with reduced connectivity strength in the Segregated compared to the Integrated state are shown here in darker colour. Key differences were in temporal and medial frontal regions.

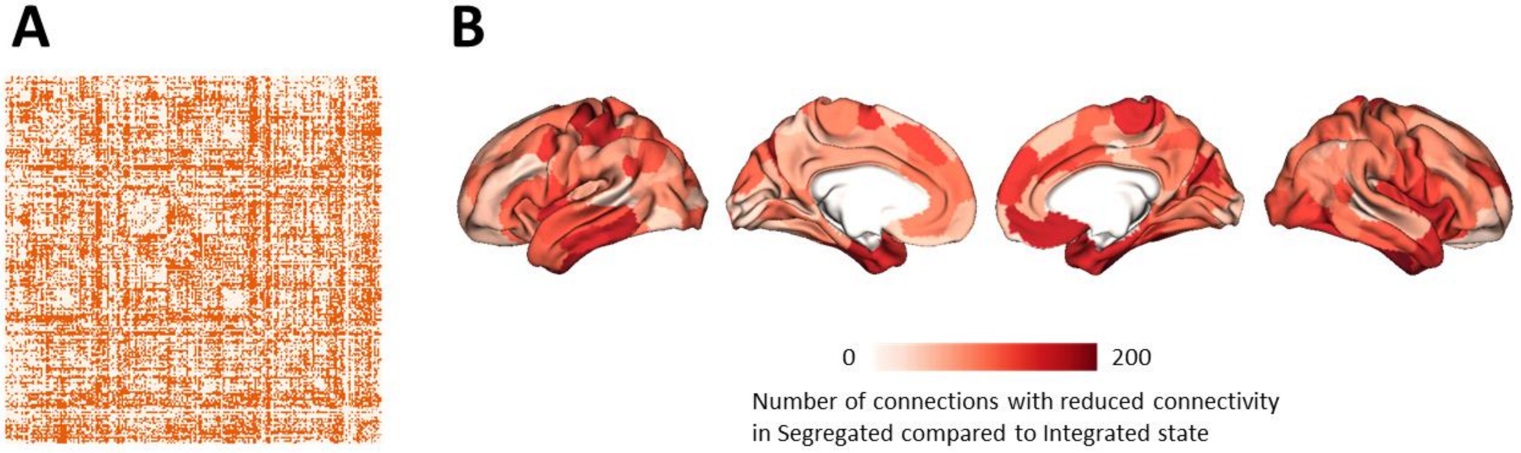

Supplementary Table 3. Differences in Integration and Segregation between Integrated and Segregated states

	Integrated state	Segregated state	p-value	q-value
**Measures of Integration**				
Characteristic path length	2.673 (2.584)	3.003 (2.284)	0.082	0.115
Global efficiency	0.958 (0.069)	0.939 (0.081)	**0.043**	0.077
Participation coefficient	0.495 (0.128)	0.456 (0.117)	**0.013**	**0.045**
**Measures of segregation**				
Clustering coefficient	0.671 (0.225)	0.692 (0.243)	0.445	0.519
Modularity	0.467 (0.118)	0.490 (0.127)	0.647	0.647
**Other measures**				
Small world propensity	0.427 (0.082)	0.104 (9.664)	**0.002**	**0.014**
Density	1.824 (0.277)	1.752 (0.319)	**0.044**	**0.077**

Results are mean (standard deviation) and p-value of Kruskal Wallis test.
q-value: FDR corrected p-value.
Note that the state with the highest average participation coefficient was chosen a-priori as the Integrated state for each participant and the one with the lowest average participation coefficient was chosen as the Segregated state.
Characteristic path length: defined as the average shortest path length across all pairs of nodes; lower values imply higher degree of integration
Global efficiency: the average of the inverse shortest path length; higher values imply higher degree of integration
Participation coefficient: measures the strength of a node's connections within its community; higher participation coefficient overall implies higher interconnectivity of the graph's nodes hence higher integration
Clustering coefficient: quantifies how much the neighbours of a given node are interconnected; higher values imply higher degree of segregation
Modularity: quantifies the degree to which the network may be subdivided into clearly delineated modules; higher values imply higher degree of segregation

Supplementary Figure 3. Mean minimal control energy to maintain and transition between sub-states

A: Across all participants, the minimal control energy that is required across the whole of the brain network (232 regions of interest) to maintain the Integrated sub-state (blue) was significantly higher than the energy needed to maintain the Segregated sub-state (green): repeated measures ANOVA main effect of integrated to segregated state persistence energy $F(1,113) = 12.432$, $p<0.001$).

B: Across all participants, the minimal energy needed to transition from the more interconnected integrated to the less connected segregated state (blue) was lower than that to transition from the segregated to the integrated state (green, ($F(1,113)= 6.722$, $p=0.011$))

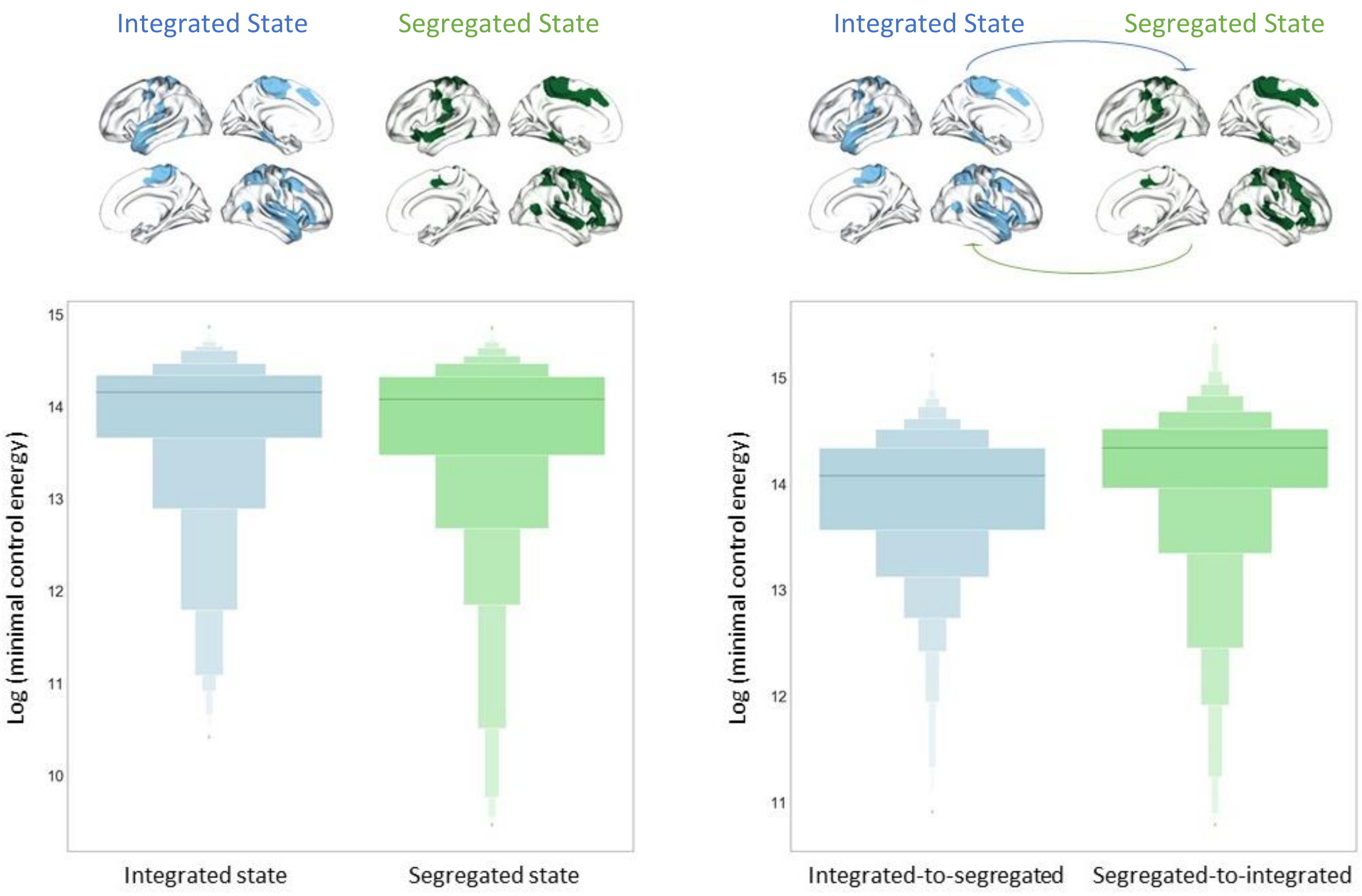

Supplementary Figure 4. Altered temporal properties of dynamic functional connectivity in patients with Parkinson’s and visual hallucinations:Replication with Schaeffer Parcellation 454

Percentage of total time spent in the Integrated state.

Patients with Parkinson’s with visual hallucinations spent significantly less time in the Integrated sub-state of dynamic functional connectivity than patients without hallucinations (p=0.032) and controls (p=0.0262).

PD-VH: Parkinson’s disease with visual hallucinations, PD non VH: Parkinson’s disease without hallucinations. UM-PDHQ: University of Miami Parkinson’s disease Hallucinations Questionnaire, higher scores indicate more severe and frequent hallucinations. Error bars are 95% confidence intervals.

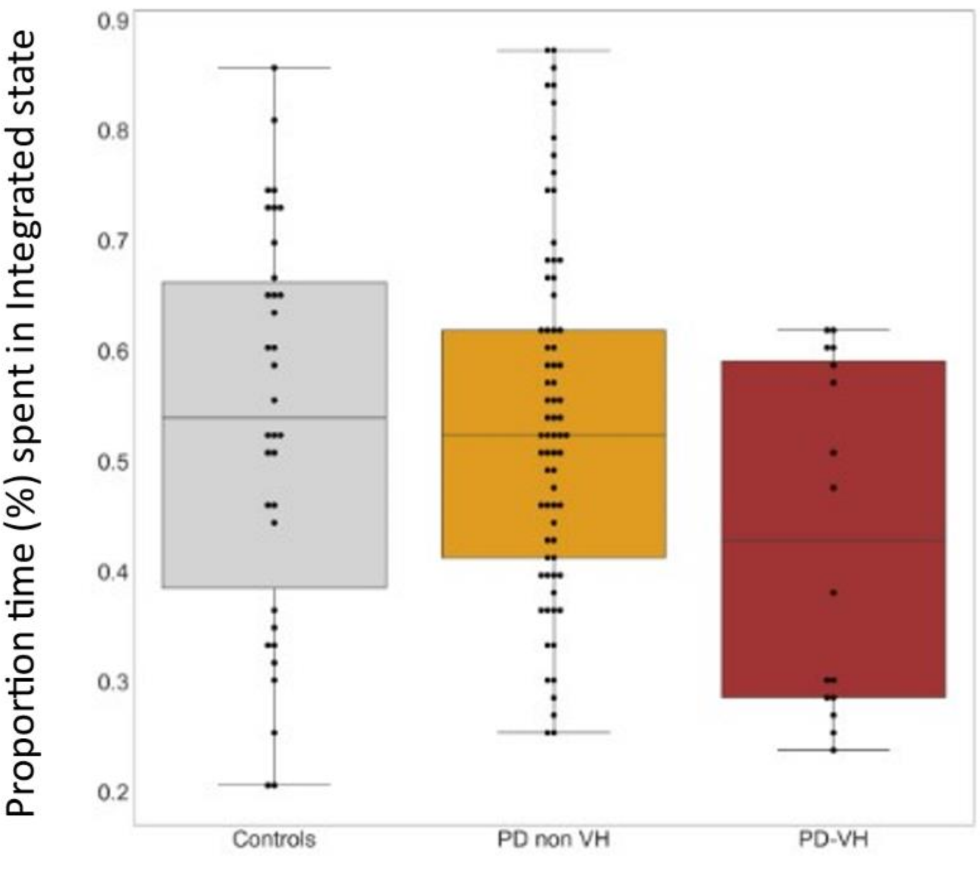

URLs

Analysis code for this study: https://github.com/AngelikaZa/TVFC

Abagen (toolbox to derive gene expression atlas in parcellation space):

https://abagen.readthedocs.io/en/stable/index.html

Brain Connectivity Toolbox: https://sites.google.com/site/bctnet/

BrainSpy (MNI coordinates check): https://github.com/ezPsycho/brainSpy-cli

Code to derive "cartographic profile": https://github.com/macshine/integration/

Code to compute small-world propensity: http://www.seas.upenn.edu/~dsb/

JuSpace (toolbox to derive neurotransmitter density profiles):

https://github.com/juryxy/JuSpace

Schaefer brain parcellation:

https://github.com/ThomasYeoLab/CBIG/tree/master/stable_projects/brain_parcellation/Schaefer2018_LocalGlobal

Spatial permutations at parcellation level:

https://github.com/frantisekvasa/rotate_parcellation
