# Supplementary material for: Changes in dynamic transitions between integrated and segregated states underlie visual hallucinations in Parkinson’s disease
Source: Commun Biol. 2022 Sep 8;5:928. doi: 10.1038/s42003-022-03903-x (PMC9458713; doi:10.1038/s42003-022-03903-x)
Supplement: Supplementary file 3 — Description of Additional Supplementary Files [file 42003_2022_3903_MOESM3_ESM.pdf]

Description of Additional Supplementary Files

File name: Supplementary Data 1
Description: Source data behind the graphs in the paper: Clinical and meta-state data per participant.

File name: Supplementary Data 2
Description: Source data behind the graphs in the paper: Minimal control energy per node for Intergated-to-Segregated transition.

File name: Supplementary Data 3
Description: PET derived neurotransmitter density per parcellation node.
