# Supplementary material for: Changes in dynamic transitions between integrated and segregated states underlie visual hallucinations in Parkinson’s disease
Source: Commun Biol. 2022 Sep 8;5:928. doi: 10.1038/s42003-022-03903-x (PMC9458713; doi:10.1038/s42003-022-03903-x)
Supplement: Supplementary file 5 — Reporting Summary [file 42003_2022_3903_MOESM5_ESM.pdf]

Corresponding author(s): Angeliki Zarkali

Last updated by author(s): 2021/08/13

Reporting Summary

Nature Portfolio wishes to improve the reproducibility of the work that we publish. This form provides structure for consistency and transparency in reporting. For further information on Nature Portfolio policies, see our Editorial Policies and the Editorial Policy Checklist.

Statistics

For all statistical analyses, confirm that the following items are present in the figure legend, table legend, main text, or Methods section.

n/a	Confirmed	
☐	☒	The exact sample size (*n*) for each experimental group/condition, given as a discrete number and unit of measurement
☐	☒	A statement on whether measurements were taken from distinct samples or whether the same sample was measured repeatedly
☐	☒	The statistical test(s) used AND whether they are one- or two-sided<br>*Only common tests should be described solely by name; describe more complex techniques in the Methods section.*
☐	☒	A description of all covariates tested
☐	☒	A description of any assumptions or corrections, such as tests of normality and adjustment for multiple comparisons
☐	☒	A full description of the statistical parameters including central tendency (e.g. means) or other basic estimates (e.g. regression coefficient) AND variation (e.g. standard deviation) or associated estimates of uncertainty (e.g. confidence intervals)
☐	☒	For null hypothesis testing, the test statistic (e.g. *F*, *t*, *r*) with confidence intervals, effect sizes, degrees of freedom and *P* value noted<br>*Give P values as exact values whenever suitable.*
☒	☐	For Bayesian analysis, information on the choice of priors and Markov chain Monte Carlo settings
☒	☐	For hierarchical and complex designs, identification of the appropriate level for tests and full reporting of outcomes
☐	☒	Estimates of effect sizes (e.g. Cohen's *d*, Pearson's *r*), indicating how they were calculated

Our web collection on statistics for biologists contains articles on many of the points above.

Software and code

Policy information about availability of computer code

Data collection	Demographic, clinical and psychometric data was collected and stored in excel. MRI data was collected using a 3T Siemens scanner and processed using Mrtrix 3.0 for diffusion data and Nipype for resting state fmri data. All steps of data processing are described in the manuscript.
Data analysis	States of dynamic functional connectivity were derived using the cartographic profile as described here: https://github.com/macshine/integration/. Energy costs for state transitions were derived using code adjusted from https://github.com/jastiso/NetworkControl. Statistical analyses were performed in Python 3 using Jupyter Lab v.1.0.2. All analyses are described in full in the manuscript.

For manuscripts utilizing custom algorithms or software that are central to the research but not yet described in published literature, software must be made available to editors and reviewers. We strongly encourage code deposition in a community repository (e.g. GitHub). See the Nature Portfolio guidelines for submitting code & software for further information.

Data

Policy information about availability of data

All manuscripts must include a data availability statement. This statement should provide the following information, where applicable:

- Accession codes, unique identifiers, or web links for publicly available datasets
- A description of any restrictions on data availability
- For clinical datasets or third party data, please ensure that the statement adheres to our policy

All data and results of statistical analyses are presented in the manuscript. Analysis code is available on github (https://github.com/AngelikaZa/TVFC). Individual level data will be made available to researchers upon request.

Field-specific reporting

Please select the one below that is the best fit for your research. If you are not sure, read the appropriate sections before making your selection.

☒ Life sciences ☐ Behavioural & social sciences ☐ Ecological, evolutionary & environmental sciences

For a reference copy of the document with all sections, see nature.com/documents/nr-reporting-summary-flat.pdf

Life sciences study design

All studies must disclose on these points even when the disclosure is negative.

Sample size	Participants were recruited to the Vision in Parkinson's disease study, a longitudinal observation study. The study was powered to be able to detect differences in high level visual performance at baseline for participants with Parkinson's disease.
Data exclusions	12 participants were excluded as they failed predetermined quality control criteria (described in the Supplementary Material) for resting state fMRI data. 7 participants were excluded as they failed quality control criteria for structural imaging and were excluded from the network control theory analysis.
Replication	We have replicated our analysis using two parcellations of different granularity (232 and 454 nodes) to ensure robustness of our results.
Randomization	This was an observational study therefore no randomisation took place.
Blinding	The researchers collecting clinical and imaging data were blinded during data collection. Groups were derived later from participant responses to clinical questionnaires and not allocated by the researchers.

Reporting for specific materials, systems and methods

We require information from authors about some types of materials, experimental systems and methods used in many studies. Here, indicate whether each material, system or method listed is relevant to your study. If you are not sure if a list item applies to your research, read the appropriate section before selecting a response.

Materials & experimental systems

n/a	Involved in the study	
☒	☐	Antibodies
☒	☐	Eukaryotic cell lines
☒	☐	Palaeontology and archaeology
☒	☐	Animals and other organisms
☐	☒	Human research participants
☐	☒	Clinical data
☒	☐	Dual use research of concern

Methods

n/a	Involved in the study	
☒	☐	ChIP-seq
☒	☐	Flow cytometry
☐	☒	MRI-based neuroimaging

Human research participants

Policy information about studies involving human research participants

Population characteristics	91 patients with PD were included in this study: 16 PD patients with habitual, at least weekly, visual hallucinations (PD-VH), 75 PD patients without hallucinations (PD-non-VH) and 32 controls. Mean age was 64.9 years (range 49 - 82), 52.0% (n=64) were male.
Recruitment	We recruited 91 patients with PD to our London centre from clinics in the National Hospital for Neurology and Neurosurgery and affiliated hospitals. All patients with PD fullfilled the Queen Square Brain Bank Criteria. We also recruited 32 unaffected controls from volunteer databases in our institution and patient spouses.
Ethics oversight	The study was approved by the Queen Square Ethics committee. REC reference: 15/LO/0476.

Note that full information on the approval of the study protocol must also be provided in the manuscript.

Clinical data

Policy information about clinical studies

All manuscripts should comply with the ICMJE guidelines for publication of clinical research and a completed CONSORT checklist must be included with all submissions.

Clinical trial registration	REC reference: 15/LO/0476

Study protocol	The study protocol and other information about the study can be found here: https://vision-in-parkinsons.co.uk/
Data collection	All clinical and imaging data was collected over a single study visit. Clinical and psychological assessments were performed at the Institute of Neurology, Queen Square, University College London. All imaging data was collected at the same scanner (3T Siemens) at the Wellcome Centre for Neuroimaging, University College London.
Outcomes	This was an observational study. Participants were classified as hallucinators if they answered more than 1 on Question 2.1 of the Unified Parkinson's Disease Rating Scale (UPDRS) (n=16). All other patients were classified as PD-non-VH (n=75). We collected additional information on severity, frequency and the phenomenology of experienced hallucinations with the University of Miami Parkinson's Disease Hallucinations Questionnaire (UM-PDHQ).

Magnetic resonance imaging

Experimental design

Design type	Resting state
Design specifications	NA
Behavioral performance measures	NA

Acquisition

Imaging type(s)	Resting state functional MRI
Field strength	3
Sequence & imaging parameters	All MRI data were acquired on a 3T Siemens Magnetom Prisma scanner (Siemens) with a 64-channel head coil. Resting state functional MRI (rsfMRI) was acquired with the following parameters: gradient-echo EPI, TR=70ms, TE=30ms, flip angle=90°, FOV=192×192, voxel size=3x3x2.5 mm, 105 volumes, 7-minute session. During rsfMRI, participants were instructed to lie quietly with their eyes closed and avoid falling asleep; this was confirmed by monitoring and post-scan debriefing. A 3D MPRAGE (magnetization prepared rapid acquisition gradient echo) image (voxel size=1×1×1 mm, TE=3.34ms, TR= 2530 ms, flip angle=7°) was also obtained. Imaging for all participants was performed at the same time of day, with PD participants receiving their normal medications.
Area of acquisition	Whole brain scan

Diffusion MRI ☒ Used ☐ Not used

Parameters	Diffusion weighted imaging (DWI) was acquired with the following parameters: b0 in both AP and PA directions, b=50 s/mm2 /17 directions, b=300 s/mm2 /8 directions, b=1000 s/mm2 /64 directions, b=2000 s/mm2 /64 directions, 2x2x2 mm isotropic voxels, TE=3260ms, TR=58ms, 72 slices, 2mm thickness, acceleration factor = 2. DWI acquisition time was approximately 10 min.

Preprocessing

Preprocessing software	Pre-processing of DWI images was performed in MRtrix3.0.<br>rsfMRI data underwent standard pre-processing using fMRIPrep 1.5.0.
Normalization	Diffusion weighted imaging were analysed at individual space. The raw T1-weighted images were registered to the diffusion-weighted image using NiftyReg and five-tissue anatomical segmentation performed using the 5ttgen script in MRtrix. Resting state fmri images were normalised to MNI space. Motion correcting transformations, field distortion correcting warp, BOLD-to-T1w transformation and T1w-to-template (MNI) warp were concatenated and applied in a single step using antsApplyTransforms (ANTs v2.1.0) using Lanczos interpolation.
Normalization template	MNI 2009 for resting state fMRI data.
Noise and artifact removal	Diffusion weighted images underwent denoising, removal of Gibbs artefacts, eddy-current and motion correction and bias field correction. Diffusion tensor metrics were calculated and constrained spherical deconvolution performed.<br>Functional data was slice-time corrected using 3dTshift from AFNI106 and motion corrected using mcflirt. Distortion correction was performed using a TOPUP implementation. This was followed by co-registration to the corresponding T1-weighted image using boundary-based registration with six degrees of freedom.<br>Physiological noise regressors were extracted applying CompCor. Sources of spurious variance were removed through linear regression (six motion parameters, mean signal from white matter and cerebrospinal fluid), followed by calculation of bivariate correlations and application of Fisher transform.
Volume censoring	For resting state fMRI, the first 4 volumes were discarded to allow for steady state equilibrium.

Statistical modeling & inference

Model type and settings	Temporal properties of the two dynamic states of functional connectivity (Integrated and Segregated states, derived from each participant's cartographic profile) were assessed by comparing mean dwell time in each state, proportion spent in

Integrated vs Segregated state and number of transitions between PD-VH vs PD-non-VH and PD vs controls. As these were not normally distributed group-differences were tested using Kruskal Wallis (post-hoc Dunn, p<0.05). Differences in transition and persistence energy between PD-VH vs PD-non-VH were performed using repeated measures ANOVA (p<0.05). Additionally we investigated whether each of the two states significantly differed across groups using network-based statistics. A general linear model was used with PD-VH versus PD-non-VH and PD versus controls as contrasts of interest and age and total intracranial volume as covariates. Permutation testing with unpaired t-tests was performed (5000 permutations), calculating a test statistic for each connection. An a-priori threshold of t=2.7 was applied based on our sample size and family-wise error rate (FWE) of p<0.05.

Effect(s) tested

We tested differences between PD-VH and PD-non-VH (primary comparison) and PD versus controls. We correlated regional control energy with 1) regional receptor density profiles for serotonin (5HT1a, 5HT2a and 5HT1b), dopamine (D1 and D2) and GABA receptors, and 2) regional gene expression for 31 pre-selected genes encoding receptors for norepinephrine, acetylcholine, dopamine and serotonin.

Specify type of analysis: ☒ Whole brain ☐ ROI-based ☐ Both

Statistic type for inference
(See Eklund et al. 2016)

Kruskal Wallis, repeated measures ANOVA, general linear model (as described above).

Correction

Multiple comparisons were corrected using Family wise error correction for Network based statistics and False Discovery Rate (Benjamini-Hochberg method, q<0.05) for neurotransmitter correlations.

Models & analysis

n/a	Involved in the study
☒	☐ Functional and/or effective connectivity
☐	☒ Graph analysis
☒	☐ Multivariate modeling or predictive analysis

Graph analysis

Functional states of higher or lower integration were derived for each participants resting state functional data using a “cartographic profile”. Dynamic connectivity matrices were derived using an overlapping sliding-window approach10 with windows of 44s duration (63*TR) in steps of 1 repetition size; resulting in 63 windows of 44s duration. Within each temporal window, we calculated a 232*232 weighted adjacency matrix representing the functional connetome for that timepoint. Then, for each participant, modules (non-overlapping groups of highly connected nodes) were identified using the data-driven Louvain algorithm. The participation coefficient and Z-score of within-module degree was calculated for each ROI at each timepoint (Supplementary Methods 4). K-means clustering was performed and assigned each dynamic functional connectivity matrix to one of two clusters. The cluster with the higher average participation coefficient was defined as the “Integrated” state and the cluster with the lower average participation coefficient as the “Segregated” state.
We then calculated: 1) proportion of time spent in each state as the number of timepoints within each state divided by number of total timepoints, 2) average dwell time as the number of consecutive windows/ timepoints belonging to each state and 3) number of transitions as the number of transitions from one state to the other; transitions were further divided into transitions from integrated-to-segregated and from segregated-to-integrated states.
Finally, we used diffusion weighted imaging and network control theory to calculate the control energy to be applied to each node of the network to: 1) transition from the integrated-to-segregated state, (using x0 (baseline state) the sum connectivity vector of the Integrated state; and xT, (target state), the sum connectivity vector of the Segregated state), 2) transition from the segregated-to-integrated state, using as x0 the sum connectivity vector of the Segregated and state xT, the sum connectivity vector of the Integrated state, and 3) persist within the Integrated or within the Segregated state (i.e. transition from one state to itself), using the sum connectivity vector for that state for both x0 and xT. A sum of the control energies to be applied across all nodes of the network represents the minimal energy for the specific transition.
